# A Tug of War: Pseudorabies Virus and Host Antiviral Innate Immunity

**DOI:** 10.3390/v14030547

**Published:** 2022-03-06

**Authors:** Guangqiang Ye, Hongyang Liu, Qiongqiong Zhou, Xiaohong Liu, Li Huang, Changjiang Weng

**Affiliations:** 1 State Key Laboratory of Veterinary Biotechnology, Division of Fundamental Immunology, Harbin Veterinary Research Institute of Chinese Academy of Agricultural Sciences, Harbin 150069, China; ygqyyds123@163.com (G.Y.); lhyyyds1234@163.com (H.L.); qqhenanly@163.com (Q.Z.); lxhyyds1234@163.com (X.L.); highlight0315@163.com (L.H.); 2 Heilongjiang Provincial Key Laboratory of Veterinary Immunology, Harbin 150069, China

**Keywords:** pseudorabies virus, innate immune response, type I interferons, apoptosis, autophagy

## Abstract

The non-specific innate immunity can initiate host antiviral innate immune responses within minutes to hours after the invasion of pathogenic microorganisms. Therefore, the natural immune response is the first line of defense for the host to resist the invaders, including viruses, bacteria, fungi. Host pattern recognition receptors (PRRs) in the infected cells or bystander cells recognize pathogen-associated molecular patterns (PAMPs) of invading pathogens and initiate a series of signal cascades, resulting in the expression of type I interferons (IFN-I) and inflammatory cytokines to antagonize the infection of microorganisms. In contrast, the invading pathogens take a variety of mechanisms to inhibit the induction of IFN-I production from avoiding being cleared. Pseudorabies virus (PRV) belongs to the family Herpesviridae, subfamily Alphaherpesvirinae, genus Varicellovirus. PRV is the causative agent of Aujeszky’s disease (AD, pseudorabies). Although the natural host of PRV is swine, it can infect a wide variety of mammals, such as cattle, sheep, cats, and dogs. The disease is usually fatal to these hosts. PRV mainly infects the peripheral nervous system (PNS) in swine. For other species, PRV mainly invades the PNS first and then progresses to the central nervous system (CNS), which leads to acute death of the host with serious clinical and neurological symptoms. In recent years, new PRV variant strains have appeared in some areas, and sporadic cases of PRV infection in humans have also been reported, suggesting that PRV is still an important emerging and re-emerging infectious disease. This review summarizes the strategies of PRV evading host innate immunity and new targets for inhibition of PRV replication, which will provide more information for the development of effective inactivated vaccines and drugs for PRV.

## 1. Introduction

Virus infection induces host innate immune responses, which play an important and decisive role in determining the outcome of the infected host, inducing acute infection death or establishing persistent infection in mammals. The host can establish an antiviral status based on innate immunity systems to antagonize the invasion of the virus by identifying the components of invading virus through pattern recognition receptors (PRRs), which activate signaling pathways to produce type I interferons (IFN-I) [1]. The released IFN-I binds IFN receptors (IFNAR1 and/or IFNAR2) to activate the transduction of downstream JAK-STAT signaling pathway, eventually leading to the expression of a variety of interferon-stimulated genes (ISGs). Accumulating data showed that the ISGs could achieve many cellular outcomes, including antiviral defense, antiproliferative activities, and stimulation of adaptive immunity [2,3,4].

In the process of host resistance to virus invasion, recognition of PAMPs by host PRRs is the first step of innate immunity [5]. For RNA virus, viral RNA is often recognized by a variety of PRRs, including endosomal Toll-like receptor 3 (TLR3), cytosolic retinoic acid-inducible gene I (RIG-I), melanoma differentiation-associated gene 5 (MDA5), and LGP2/DHX58 sense viral RNA, NOD-like receptor protein 3 (NLRP3), nucleotide-binding oligomerization domain containing 2 (NOD2) [6,7]. For DNA virus, Toll-like receptor 9 (TLR9), cyclic GMP-AMP (cGAMP) synthase (cGAS), DAI (DLM-1/ZBP1), absent in melanoma 2 (AIM2), and IFN gamma-inducible protein 16 (IFI16) act as the main PRRs that recognize viral DNA [8,9]. PRRs then recruit a series of important signal transduction molecules, such as myeloid differentiation primary response gene 88 (MyD88), mitochondrial antiviral-signaling protein (MAVS), intracellular stimulator of IFN genes (STING). These proteins then transfer the different signals to the downstream molecules in different signaling pathways, which eventually lead to activation and translocation of several transcription factors, including NF-κB, interferon regulatory factor 3 (IRF3), and IRF7 into the nucleus to induce the expression of IFN-I and proinflammatory cytokines [10,11,12].

PRV, a member of alpha-herpesvirus, is the pathogen of Aujeszky’s disease. Domestic pigs and wild boars are considered the natural host of the disease, but the disease also threatens most mammals [13,14,15,16]. The persistence of PRV infection is recognized only in the family Suidae due to establishing the latent infection. Acute and lethal infection in Suidae occurs in piglets. For other species, PRV mainly invades the PNS first but then progresses to the CNS, which leads to acute death of the host with serious clinical and neurological symptoms [17]. Although humans infected with PRV do not cause death, it causes strong neurological symptoms [18,19]. Recently, as an important emerging and re-emerging infectious disease, PRV cases have been frequently reported. Therefore, PRV is still an important pathogen in agriculture. Previous studies have shown that PRV is an ideal model for studying virus escape from host immune responses in different species [17,20]. The virus is also used as a "live" tracer of a neuronal pathway because it has a significant tendency to infect synaptically connected neurons [21].

IFN-I and tumor necrosis factor (TNF) play a central role in host antiviral immunity. However, excessive production of IFN-I and TNF will cause harmful immune effects. Therefore, IFNs, TNF, and activation of their downstream are strictly regulated. On the other hand, PRV has evolved various mechanisms to inhibit the induction of IFN-I from avoiding being cleared by the active antiviral innate immunity. Accumulated evidence has shown that several alpha-herpesvirus-encoded proteins can antagonize host antiviral innate immune responses by inhibiting IFN-I production, blocking downstream IFN signaling, or regulating the specific ISGs [22,23,24,25,26].

Recently, increasing reports indicate that PRV has evolved various mechanisms to antagonize host immune responses for efficient infection. This review summarizes the immune escape strategies of PRV, including inhibition of IFN-I production and IFN signaling, modulation of inflammatory responses, regulation of apoptosis, and autophagy. These important research advances summarized in this review will help readers understand the latest PRV immune escape strategies, which will help PRV researchers design new effective vaccines and develop new antiviral drugs to prevent and control PRV.

## 2. PRV Virion

**Figure 1:**
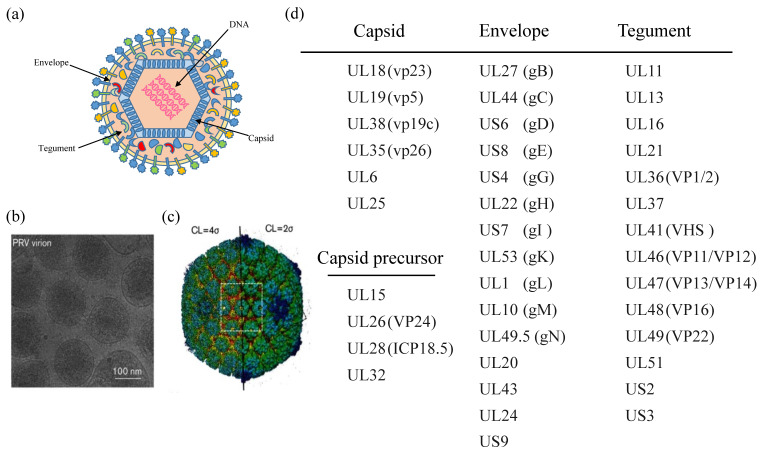
Structure and associated proteins of pseudorabies virus (PRV) virion. (**a**) Structure diagram of PRV virion. (**b**) CryoEM image of PRV virions, adapted from [30]. (**c**) 3D reconstruction of the PRV virion at 4.9 Å, colored radially, adapted from [30]. The left and right halves of the capsid are rendered at a contour level (CL) of four and two times standard deviations above the mean density (σ), respectively. The white box and the black dashed box demark a hexon and a vertex region containing five CATCs, respectively [30]. **(d**) Distribution of some proteins.

All herpesvirus virions have similar virus particle size (200–250 nm) and structure, containing a double-stranded DNA (dsDNA) genome. In morphology, the complete PRV virions are round or oval, with 150–180 nm diameter. PRV virions are composed of four structural components (Figure 1): the central core containing the viral genomic DNA is packaged in the nucleocapsid; the capsid is embedded by tegument composed of a protein matrix; the envelope is a lipid membrane containing several viral glycoproteins. Previous studies showed that the structural components of mature virus particles are composed of nearly half of PRV gene products [17]. Like VZV, the PRV genome has two unique regions (UL and US). The US region is flanked by the internal and terminal repeat sequences. Most PRV proteins have orthologs in other alpha-herpesvirus. Generally, each gene’s name and its corresponding PRV protein can refer to the location of the homologous protein in the region of the HSV-1 genome [17]. In Figure 1, some PRV-encoded proteins are listed that partially make up viral particles. However, not all proteins are shown in the figure. It should be noted that some HSV viral proteins are multifunctional proteins involved in antiviral innate immunity. For example, Huang et al. reported that HSV-1 VP22 counteracts the cGAS/STING-mediated IFN production by inhibiting the enzymatic activity of cGAS [27]. Maruzuru et al. reported that HSV-1 VP22 interacts with AIM2 and prevents its oligomerization [28]. Deschamps et al. reported that HSV-1 UL46 blocks STING-mediated IFN production signaling pathways by eliminating STING and IFI16 [29]. However, whether the homologs of PRV are functionally equivalent to these HSV-1 tegument proteins in immune evasion is still unclear.

## 3. PRV Entry

The entry of PRV virions into cells is mediated by several viral glycoproteins [31]. The attachment process of PRV virions is mediated by the interaction between glycoprotein gC and heparan sulfate proteoglycans in the extracellular matrix of the permissive cells. PRV gD is also involved in viral entry by stabilizing the virus-cell interaction. Subsequently, PRV gB, gH, and gL mediate the viral membrane fusion process by fusing the envelope of virions with the plasma membrane, resulting in the penetration of virus capsid and envelope into the cytoplasm [32]. After membrane fusion, PRV capsid binds to microtubule-like structures to be transported to the nuclear pore [33]. Subsequently, the envelope proteins (UL11, UL47, UL48, and UL49) are rapidly separated from the capsid. Finally, intracellular PRV genomic DNA is released from the morphologically intact capsid into the host nucleus and replicated. In this process, DNA located in the nuclear triggers the host antiviral immune responses and transmits the signals to the cytoplasm.

## 4. Viral DNA Are Recognized by DNA Sensors

It is well known that sensing viral DNA and activating its downstream cascades play core roles in host antiviral innate immunity. At present, more than 20 DNA sensors have been identified. TLR9, AIM2, cGAS, and IFI16 are reported for recognizing viral DNA. TLR9 is the first identified DNA sensor to recognize endogenous damaged or exogenous pathogenic DNA on the endoplasmic membrane, especially the CpG DNA motif of unmethylated bacteria [34]. AIM2 and IFI16 are the two most intensive studied AIM2-like receptors (ALRs) family members [35]. AIM2 recognizes double-stranded viral DNA and then is assembled into AIM2 inflammasome to activate inflammatory response [36]. AIM2 binds double-stranded DNA through its hematopoietic IFN-inducible nuclear protein (HIN) domain, then recruits downstream caspase-1 and adaptor apoptosis-associated speck-like protein (ASC) by pyrin domain (PYD) to assemble AIM2 inflammasome, resulting in the release of mature IL-1ꞵ/IL-18 and pyroptosis due to the cleavage of gasdermin D (GSDMD) [37,38]. IFI16 can play a variety of functions in inflammatory responses and IFN-I responses. IFI16 senses the Kaposi sarcoma-associated herpesvirus (KSHV) infection and induces the activation of inflammasomes [39]. IFI16 can recognize HSV DNA and participate in IFI16-STING and NF-κB signaling pathways [40]. Recently, cGAS was defined as a dsDNA sensor [41]. cGAS recognizes cytoplasmic dsDNA to produce secondary messenger cGAMP. Subsequently, STING located on the endoplasmic reticulum is polymerized and translocated to the Golgi, where TBK1 and IRF3 are recruited and activated to phosphorylated IRF3, the dimerized IRF3 is translocated into the nucleus, resulting in the transcriptional induction of IFN-I and the NF-κB-dependent expression [42,43,44]. The TLR signal transduction adaptor protein TRIF is also reported to be involved in the cGAS-STING signaling pathway [45]. For PRV, it has been reported that DNA-dependent activator of interferon (IFN)-regulatory factors (DAI), a DNA sensor of the PRV genome, triggers the production of IFN-I [46]. DEAD (Asp-Glu-Ala-Asp) box polypeptide 41 (DDX41), a member of the DEXDc helicase family, also has been identified as another intracellular DNA sensor of the genomic DNA of PRV in porcine kidney cells [47]. Subsequently, Wang et al. also demonstrated that cGAS senses cytosolic PRV DNA and promotes IFN-β expression [48]. However, why cells use different DNA sensors to defend against PRV infection and the coordinated roles of these PRRs in recognizing the PRV genome remain unknown.

## 5. PRV Infection Inhibits IFN-I Production

**Figure 2:**
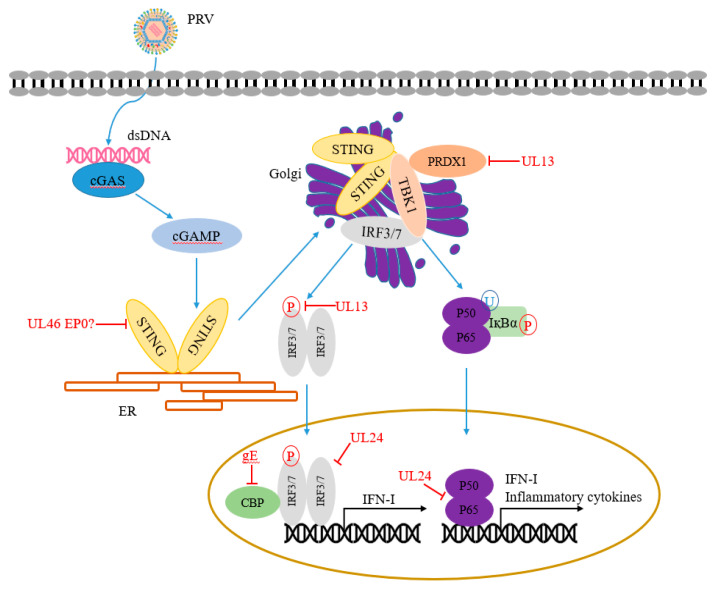
PRV evades the host antiviral immune responses by regulating the DNA-sensing signaling pathway. PRV invasion induces type I interferons (IFN-I) production through IRFs or NF-κB signal pathways. PRV-encoded multiple proteins can target various steps involved in this process.

This section summarizes the PRV-encoded proteins that participate in regulating NF-κB and cGAS-STING signaling pathways (Table 1 and Figure 2).

### 5.1. cGAS

During PRV infection, cGAS senses cytosolic PRV DNA to synthesize cGAMP and then activate the STING/TBK1/IRF3 signaling pathway to produce IFN-I [48]. Histone deacetylases (HDACs) are epigenetic regulators that regulate the histones, chromatin conformation, protein-DNA interaction, and even transcription. Recently, Guo et al. found that genetic and pharmacological inhibition of HDAC1 significantly influences PRV replication in that the inhibition of HDAC1 induces DNA damage response, resulting in the release of damaged DNA into the cytosol, then activates cGAS and the downstream STING/TBK1/IRF3 signaling pathway [61]. Walters et al. found that the *US3* gene of PRV is conserved in HSV-1 and varicella-zoster virus (VZV). US3 is a serine/threonine (S/T) kinase. HDAC2 is hyperphosphorylated in cells infected with PRV and PRV lacking US3 kinase. However, specific chemical inhibition of class I HDAC activity increases the plaquing efficiency of PRV lacking US3 kinase, whereas only minimal effects are observed with wild-type viruses, indicating that PRV US3 kinase activity is required for HDACs to reduce viral genome silencing and allow efficient viral replication [62].

### 5.2. STING

STING, an evolutionarily conserved transmembrane protein [63], plays a central role in antiviral immunity by activating its downstream TBK1-IRF3 axis [64]. A variety of viruses has evolved many strategies to target STING, resulting in the inhibition of innate immune responses. For example, HSV ICP34.5 interacts with STING and prevents its transport from the endoplasmic reticulum to Golgi, inhibiting host innate immunity [65]. PRV *UL46* gene is a late gene that encodes viral phosphoproteins 11 and 12 (VP11/12). Recently, PRV UL46 has been shown to induce phosphorylation of ERK1/2, which is not involved in impairing the integrity of the nuclear envelope [66]. Xu et al. found that PRV UL46 is a nucleocytoplasmic shuttling tegument protein. UL46 interacts with EP0, UL48, and STING [67]. However, whether these interactions affect STING-mediated host innate immune responses remain unknown.

### 5.3. TBK1

Tank binding kinase 1 (TBK1) is a serine/threonine kinase involved in various biological processes, including innate immune response, autophagy, and cell growth [68]. In the long-term struggle between host and virus, as a key adaptor protein of multiple signal pathways [69,70,71], TBK1 has become the target of many hosts’ immune regulatory factors and viral proteins.

PRV UL13 is a protein serine/threonine kinase, which can be packaged into the tegument of PRV virions. Lv et al. found that peroxiredoxin 1 (PRDX1), a member of antioxidant enzymes, binds to TBK1 and IκB kinase ε (IKKε) to regulate IFN-I production positively. Studies have shown that UL13 interacts with and promotes antiviral regulator PRDX1 degradation via the ubiquitin-proteasome pathway in a kinase-dependent manner, thereby inhibiting the host’s innate immune response [49].

### 5.4. UL13, UL24, US3, and gE Target IRF3/IRF7

IRF3 plays a key role in the induction of IFN-I production. As the central factor of antiviral response, IRF3 is targeted by many viral proteins through ubiquitination and phosphorylation. PRV has evolved a variety of antiviral strategies to antagonize IRF3 function. Recently, LV et al. found that PRV protein kinase UL13 inhibits IFN-I production. by enhancing the ubiquitination and degradation of IRF3 [50]. Bo et al. showed that PRV UL13 phosphorylates IRF3 and disrupts the interaction between IRF3 and the IRF3-responsive promoter, thereby inhibiting cGAS-STING signaling [51].

UL24 protein is a conserved protein in the herpesvirus family, required for viral growth. HSV-1 UL24 is a nucleolar protein, and the endonuclease motif of UL24 is required for viral diffusion [72,73]. HSV-1 UL24 inhibits cGAS-STING-mediated NF-κB promoter activity, dependent on the region containing 74 to 134 aa within HSV-1 UL24. Mechanistically, HSV-1 UL24 binds to NF-κB subunits p65 and p50 via the Rel homology domains (RHDs), which reduces the tumor necrosis factor-alpha (TNF-α)-mediated nuclear translocation of p65 and p50 [74]. Liu et al. showed that PRV UL24 promotes IRF7 degradation through the ubiquitin-proteasome pathway, resulting in antagonizing cGAS-STING-mediated the production of IFN-I [52].

US3 is a conserved serine/threonine kinase in all herpes viruses. It plays an important role in virus replication. It has been reported that PRV US3 is mainly involved in the nuclear export of viral capsids, which is related to the pathogenicity of the virus in vivo [75]. On the other hand, PRV US3 induces cytoskeleton changes in PRV-infected cells [76,77] and has different effects on various host defense mechanisms. Xie et al. demonstrated that PRV US3 inhibits IFN-I production by degrading IRF3. The results showed that PRV US3 could inhibit host antiviral innate immunity by targeting various host defense mechanisms [56]. CREB-binding protein (CBP/p300) is a histone acetyltransferase, which has been proved to play an important role in transcriptional regulation. Previous studies showed that CBP/p300 could interact with IRF3, NF-κB, p53, and other transcription factors [78,79]. Lu et al. found that PRV glycoprotein gE suppresses IFN-β production by targeting IRF3 and promotes CBP/p300 degradation [58].

Recently, several studies have confirmed that PRV has also evolved a variety of immune escape strategies in plasma cells, such as dendritic cells (pDCs). pDCs play a central role by producing many IFN-I in the antiviral immune response [80]. Lamote et al. showed that the lack of gE could enhance the phosphorylation of foreign signal-regulated kinase 1/2 (ERK1/2) in PDCs, thereby inducing high levels of IFN-I in pDCs. gE/gI glycoprotein complex was identified as an inhibitor of pDCs activity [81].

### 5.5. NF-κB Signaling Pathway

NF-κB family members play important roles in many biological processes, such as immunity, inflammation, and cell proliferation. The NF-κB transcription factor can be activated by various cell stimulants. NF-κB interacts with key adaptor proteins, which eventually leads to induce the expression of IFN-I and proinflammatory cytokines to execute its antiviral function [82,83,84]. PRV proteins can inhibit the transduction of the NF-κB signaling pathway in the process of infection. Wang et al. demonstrated that PRV UL24 promotes p65 degradation through the ubiquitin-proteasome pathway to eliminate TNF-α-mediated activation of NF-κB [53]. Recently, Romero et al. demonstrated that PRV infection induces DNA damage response (DDR), which then activates NF-κB via a peculiar “inside-out” nucleus-to-cytoplasm signal. The DDR-NF-κB signaling axis requires the expression of viral proteins but is initiated before active PRV replication. However, late PRV proteins inhibit NF-κB-dependent gene expression [85].

## 6. PRV Infection Inhibits IFN Signaling Pathway

**Figure 3:**
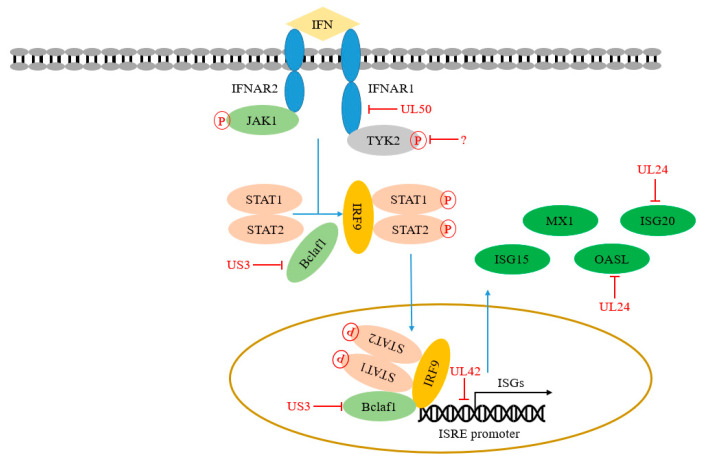
PRV infection inhibits IFN signaling pathway. The secreted IFN-I binds to IFNAR1 and/or IFNAR2 on the cell surface and initiates the transcription of hundreds of antiviral factors ISGs through the JAK-STAT signal pathway. PRV-encoded multiple proteins can target various steps involved in this process.

The secreted IFN-I binds to IFNAR1 and/or IFNAR2 to activate JAK1 and Tyk2, which then phosphorylates STAT1 and STAT2 and forms a trimer together with IRF9, called IFN stimulating gene factor 3 (ISGF3). ISGF3 then binds to IFN stimulating response element (ISRE) in the nucleus and initiates the transcription of hundreds of ISGs [86]. Some of these ISGs-encoding proteins are directly involved in antiviral response. Of course, the JAK-STAT signaling pathway plays an important role in inhibiting PRV replication and transmission. On the contrary, PRV has evolved various immune escape strategies against the JAK-STAT signaling pathway. Here, we list the key adaptor proteins in the JAK-STAT signaling pathway and summarize the immune escape strategies of PRV observed in the past five years (Table 1 and Figure 3).

### 6.1. UL50 Regulates IFNARs

IFN-I binds IFNARs (IFNAR1 and/or IFNAR2) is the initial step of the JAK-STAT signaling pathway. As the important membrane receptors, IFNARs play important roles in the immune process of the host. Consistently, patients with IFNAR1 deficiency are prone to severe COVID-19 [87], indicating that IFNAR1 plays a central role in host innate immunity. Previous studies showed that IFNAR1 is necessary for IFN signaling transduction, while IFNAR2 is necessary for STAT binding and activation. Shemesh et al. proposed that IFNAR2 is a platform for STAT activation. Interestingly, tyrosine phosphorylation of IFNAR2 may enhance JAK-STAT signal transduction by promoting the separation of activated STAT and IFNAR2 [88].

PRV has evolved an immune escape strategy by targeting IFNARs. Zhang et al. found that PRV UL50, a dUTPase, has the function of inhibiting the JAK-STAT signaling pathway. PRV UL50 degrades IFNAR1 through the lysosomal pathway to prevent STAT1 phosphorylation induced by IFN-I [59]. However, PRV UL50-mediated inhibition effect does not depend on its dUTPase activity. Its functional region for inhibiting the JAK-STAT signaling pathway corresponds to 225 to 253 aa in its C-terminal region.

### 6.2. PRV Infection Degrades JAK1 and Tyk2

Yin et al. found that PRV infection can lead to the degradation of JAK1 and Tyk2 through proteasome [89], inhibiting STAT1 phosphorylation and finally interfering with JAK-STAT signal transduction. However, the early viral protein EP0 does not relate to this inhibition, suggesting that other PRV proteins might recruit other E3 ligases to execute its degradation function.

### 6.3. US3 and UL42 Regulate ISRE

ISGF3 complex binding to the ISRE promoter is a crucial link in the JAK-STAT signaling pathway, leading to the induced expression of hundreds of ISGs. Bclaf1 (Bcl-2 related transcription factor 1) was originally found as a binding protein of adenovirus E1B 19k protein [90]. Bclaf1 exerts its functions in various biological processes, including apoptosis, cancer process, and autophagy [90,91,92,93]. Recently, Qin et al. reported that Bclaf1 is an important regulator in IFN signaling during PRV infection. Bclaf1 enhances the phosphorylation of STAT1 and STAT2 in response to IFNα. Additionally, Bclaf1 facilitates ISGF3 complex binding with ISRE, improving efficient gene transcription by directly interacting with ISRE and STAT2. The two strategies actively regulate the induction of IFN production induced by PRV. Subsequently, they found that US3 of PRV and HSV-1 can degrade Bclaf1 through proteasome pathway to resist its antiviral response [57]. In addition, Zhang et al. found that PRV UL42 competes with ISG factor 3 (ISGF3) by binding to ISRE to inhibit the transcription of the related ISGs. The four conserved DNA binding sites of PRV UL42 are necessary for its inhibitory effect on the JAK-STAT signaling pathway [60].

### 6.4. UL24 Regulates ISGs

ISGs can be induced and released in an IFN-dependent or independent manner during PRV infection. ISGs help the host build their antiviral status to block viral invasion. Several viruses have evolved strategies to target these ISGs to establish various immune evasion directly. For example, oligoadenylate synthase (OAS), an ISG family member, produces 2’–5′ oligoadenylate to trigger viral RNA degradation by recruiting/binding RNase L. It has been reported that oligoadenylate synthase-like (OASL) protein plays a role in regulating host innate immunity by inhibiting cGAS-induced IFN production and enhancing RIG-I-mediated IFN induction, respectively. Recently, Chen et al. showed that OASL promotes RIG-I-mediated IFN expression, resulting in the induction of ISGs to inhibit PRV proliferation. Of note, PRV UL24 reduces the transcription of OASL, thereby damaging the RIG-I signal pathway and antagonizing the antiviral effect of OASL [54]. Chen et al. recently reported that ISG20, a member of the ISGs, can upregulate the expression of IFN-ꞵ to resist PRV infection. Further studies found that PRV UL24 inhibits the transcription of ISG20, which weakens the antiviral function of ISG20 [55]. Recently, IFN-inducible transmembrane proteins (IFITM1, 2, 3) are found to work as important host self-restriction factors, possessing a broad spectrum of antiviral effects. Xie et al. IFITM2 are crucial for controlling PRV infection by interfering with cell binding and entry [94]. However, Wang et al. found that knockdown of IFITM2 and IFITM3 expression don’t influence PRV infection. Knockdown of IFITM1 expression using RNA interference enhances PRV infection while overexpression of IFITM1 has the opposite effect, indicating that IFITM1 is required to inhibit PRV entry [95]. Whether PRV proteins antagonize the functions of IFITMs has not been reported.

## 7. Inhibition of Intrinsic Antiviral Immunity by PRV

As a specific part of innate immunity, intrinsic antiviral immunity directly restricts viral replication and assembly. Unlike PRRs indirectly inhibit viral infection by inducing interferons and other antiviral molecules, intrinsic antiviral immunity factors recognize specific viral components and directly prevent virus replication without induction of antiviral gene expression [96,97]. The nuclear domain 10 (ND10) complex consists of three key components, namely, promyelocytic leukemia antigen (PML), speckled protein of 100 kDa (Sp100), and human death domain-associated protein 6 (hDaxx) [98,99]. Yu et al. found that promyelocytic leukemia nuclear bodies (PML-NBs) inhibit PRV infection by directly engaging in the repression of viral gene transcription [100]. Everett et al. found that PRV EP0 induces a reduction in the SUMO-modified forms but not the other isoforms of PML. PRV EP0 also causes a reduction in the numbers of hDaxx foci and also in their apparent fluorescence intensity [101]. A previous study showed that HSV-1 ICP0 also induces degradation of PML and Sp100 [102,103], suggesting that herpesvirus has evolved strategies to destroy the ND10 complex to inhibit intrinsic antiviral immunity.

Recently, a study demonstrated that cholesterol 25-hydroxylase (CH25H) catalyzes the conversion of cholesterol to 25-hydroxycholesterol (25 HC), which inhibits the growth of PRV in vitro subject to its inhibitory effect on PRV attachment and entry [104]. Li et al. found that p53, a key cellular transcription factor, positively regulates viral replication and pathogenesis, providing a novel target for intrinsic host cell immunity during PRV infection [105]. However, whether PRV proteins inhibit their antiviral function is still unknown.

## 8. Regulation of Inflammatory Responses by PRV

Inflammatory cytokines, such as interleukin 1ꞵ(IL-1ꞵ) and IL-18, play roles in clearing various invading pathogens [106]. The host’s inflammatory response is regulated by a multiprotein complex called the inflammasome. An inflammasome comprises a sensor protein, ASC, and an inflammatory pro-caspase-1 [107]. Nod-like receptors (NLRs), AIM2-like receptors (ALRs), and pyrin can be used as a sensor protein, the starting point of inflammasome assembly [107]. For example, as an NLRP3 inflammasome, NLRP3 recruits ASC and pro-caspase-1, resulting in pro-caspase-1 self-cleavage to form active caspase-1 (p10/p20 tetramer). Active caspase-1 cleaves the pro-IL-1ꞵ and pro-IL-18 to secrete mature IL-1ꞵ and IL-18 [107].

Previous studies demonstrated that PRV infection activates several inflammasomes [108]. Laval et al. found that PRV infection primes peripheral nervous system (PNS) neurons to an inflammatory state regulated by TLR2 and IFN-I signaling and induces a lethal inflammatory response in vitro and in vivo [109,110]. Ye et al. found that PRV infection can activate NLRP3-mediated inflammatory responses and significantly increase the pathogenicity of PRV in infected mice [111]. Sun et al. found that PRV infection upregulates the expression level of NLRP3, pro-caspase-1, GSDMD, pro-IL-1β, and pro-IL-18, resulting in activation of NLRP3-mediated inflammatory responses and pyroptosis by cleaving GSDMD [112].

Many studies demonstrated that some drugs could reduce the inflammatory responses induced by PRV. Liu et al. confirmed that luteolin reduces the inflammatory levels in leukemia cells and mouse macrophage (RAW264.7) during PRV infection, which is manifested in inhibiting their expression levels of proinflammatory mediators, inflammatory cytokines, and their regulatory genes, iNOS and COX-2 [113]. Vitamin A [114], Dunaliella salina alga extract [115], β-carotene [116], Resveratrol [117], and Ethyl acetate fraction of flavonoids from Polygonum hydropiper L [118] have been shown to regulate the inflammatory response induced by PRV.

## 9. Regulation of Apoptosis, Autophagy, ER Stress, and Stress Granules by PRV

### 9.1. Apoptosis

Apoptosis, also named programmed cell death, is characterized by cell shrinkage, membrane blebbing, the formation of apoptotic body, and nuclear DNA fragments, which can maintain the homeostasis of host cells [119,120]. As a cellular defense mechanism, apoptosis plays an important role in preventing virus transmission and diffusion in the early stage of virus infection [121], while apoptosis enhances virus replication and egress in the later stage of viral infection.

Several studies demonstrated that PRV infection induces apoptosis. Cheung et al. found that PRV infection undergoes apoptosis with several apoptotic characteristics, including the externalization of membrane phospholipid phosphatidylserine, the activation of caspase 3, cellular DNA degradation, and morphological changes of the nucleus [122]. Lai et al. confirmed that PRV infection induces expression of proapoptotic Bcl family proteins in PK15 cells in a dose- and time-dependent manner and trigger apoptosis. PRV infection can cause oxidative stress and free radicals, cause DNA damage, and trigger apoptosis [123]. Further studies found that caffeine, a known DNA damage inhibitor, can protect cells from PRV-induced apoptosis. Antioxidant *N*-acetyl-l-cysteine can prevent the production of reactive oxygen species (ROS) in cells and protect DNA from cutting.

Interestingly, Alemañ et al. pointed out that obvious pathological changes were observed around PRV-infected neurons, but the morphological or histochemical evidence of apoptosis was not observed. However, apoptosis was easily detected among infiltrating immune cells around PRV-infected neurons [124]. Therefore, they concluded that the apoptosis of trigeminal ganglionic neurons might be blocked during PRV acute infection, while the apoptosis of infiltrating immune cells is observed during PRV infection, indicating an important mechanism of immune evasion for the PRV. Yeh et al. found that PRV infection increases the expression levels of TNF-α and its receptor. The inhibitors of p38 and JNK/SAPK can significantly reduce the numbers of PRV infection-induced apoptosis, and the expression up-regulation of TNF-α was also inhibited in this process. So they proposed that TNF-α mediates apoptosis via the activation of p38 MAPK and JNK/SAPK signaling during PRV infection [125].

PRV has evolved many strategies to block the apoptotic signaling pathway in the long-term struggle between the virus and the hosts. For example, PRV US3 can block PRV-induced apoptosis. Chang et al. demonstrated that PRV-induced apoptosis in swine-testicle (ST) cells could be inhibited by US3 in the late stage of infection, depending on its enzyme activity [126]. Chang et al. subsequently confirmed that PRV infection could increase the expression of anti-apoptotic signaling molecules, including Akt, PDK-1, and IκBα in the trigeminal ganglion. Inhibiting the Akt and NF-κB pathways in the early stage of PRV infection can promote cell death [126]. A long isoform US3 of PRV can protect ST cells from PRV- or staurosporine-induced apoptosis. The study also pointed out that US3 is located on the mitochondrial and played an important role in inhibiting PRV-induced apoptosis [127]. Consistent with these results, a PRV-ΔUS3, a recombinant virus with deletion of *US3* gene, induced more apoptosis cells, and the virus titers were lower than wild-type PRV infection. Q-VD-OPh, a broad-spectrum caspase inhibitor, inhibited apoptosis of ST and HEp-2 cells induced by PRV-ΔUS3 or WT PRV [128].

### 9.2. Autophagy

Autophagy is a conserved lysosomal degradation process involved in a mechanism for cells to maintain homeostasis [129], which the host can use to antiviral and recover damaged organelles [130]. In some cases, autophagy is necessary for cells to antagonize viral replication. It has been reported that intracellular autophagosomes can be activated to target and degrade some viral proteins during HSV-1 infection [131]. Concerning HSV-1, many studies revealed its escape strategies and the potential protective effects of autophagy during infection [132,133]. However, VZV lacks these specific genes encoding proteins interfering with autophagy, suggesting that in some cases, VZV may be able to exploit autophagy for its replication [134,135].

The relationship between PRV replication and autophagy has been reported. Previous studies showed that PRV infection induces autophagy in vitro through the classical Beclin-1-ATG7-ATG5 pathway, resulting in increased PRV replication [71]. Xu et al. reported that PRV infection induces transformation of light chain 3 (LC3-I) autophagosomes in mouse neuron 2A (N2a) cells [136], indicating that PRV infection can induce autophagy in this cell line completely. It is an obvious paradox that PRV infection-induced autophagy enhances viral replication in that autophagy antagonizes viral replication as part of the host immune response [131]. Based on their results, Sun et al. put forward a new viewpoint on the relationship between autophagy and PRV replication. They found that PRV can induce autophagy in the early stage of infection. Upon PRV replication, several PRV-encoded proteins inhibit the level of autophagy, leading to an increase in the titer of PRV. Mechanistically, US3 protein reduces the PRV-infected cells’ autophagy level by activating the AKT/mTOR pathway. In other words, PRV infection has a dual effect on the autophagy process of host cells [137].

Bcl2-associated athanogene 3 (BAG3) is first identified as BCL-2 binding protein, belonging to the BAG protein family [138], which has a highly conserved BAG domain at the C-terminal [138,139]. Lyu et al. found that BAG3 is involved in the autophagy process during PRV infection, negatively regulating virus replication. Recently, a study showed that overexpressed UL56 induces BAG3 degradation by using its C-terminal [140]. However, BAG3 degradation was not observed in the wild-type PRV (WT) or a PRV-ΔUL56 recombinant virus with deletion of the *UL56* gene; therefore, whether the occurrence of BAG3 degradation mediated by UL56 in the PRV infection process needs further study.

Based on the progress of PRV infection-induced autophagy, some therapeutic targets and drugs against PRV infection have been reported. Xing et al. found that PRV infection inhibits the AKT/mTOR signaling pathway while Platycodon grandiflorus polysaccharide (PGPS) upregulates the mTOR signaling pathway [141]. The findings showed that PGPS could inhibit viral replication by promoting autophagy. Recently, Ming et al. assessed some deubiquitinases (USPs) inhibitors’ inhibitory effect on PRV replication [142]. Among them, USP14 inhibitor b-AP15 exhibits the most dramatic. Consistently, replenishment of USP14 in USP14 null cells restored viral replication. Inhibition of USP14 induces the K63-linked ubiquitination of PRV VP16, where USP14 directly binds to ubiquitin chains on VP16 through its UBL domain during the early stage of viral infection. Mechanistically, b-AP15 induces VP16 degradation through SQSTM1/p62-mediated selective autophagy, which is related to the EIF2AK3/PERK- and ERN1/IRE1-mediated signaling pathways [142]. Interestingly, pretreatment of mice with b-AP15 activates ER stress and autophagy, resulting in inhibition of PRV infection in vivo [142]. These results showed that USP14 might be a potential therapeutic target to treat alpha-herpesvirus-induced infectious diseases.

### 9.3. ER Stress

The endoplasmic reticulum (ER) is involved in protein synthesis, folding, transportation, and secretion. It has been reported that intracellular stress states will be sensed by ER, such as heat shock, viral and bacterial infection, hypoxia, and misfolded or unfolded proteins to activate the unfolded protein response (UPR) to restore ER and cell homeostasis [143]. During viral infection, the accumulation of viral proteins can cause stress in the ER and trigger the unfolded protein response (UPR) to restore ER homeostasis [144]. Yang et al. reported that the expression of glucose-regulated protein 78 (GRP78), as a marker of ER stress, is upregulated in the early stage of PRV infection, indicating that PRV infection induces ER stress and unfolded protein response (UPR). In addition, the IRE1-XBP1 and eIF2α-ATF4 pathways are activated during PRV infection [145]. Whether PRV proteins regulate ER stress is still unknown. Upon HSV-1 infection, HSV-1 UL41 protein suppresses the IRE1/XBP1 signaling pathway of the UPR via its RNase activity. Ectopic expression of HSV-1 UL41 decreases the expression of XBP1 and blocks XBP1 splicing activation induced by the ER stress inducer thapsigargin. Compared with HSV-1, the HSV-1 mutant lacking the *UL41* gene did not induce the decreased XBP1 mRNA induced by thapsigargin [144]. Only ATF6 activation is detected during early infection while the activity of the eIF2alpha/ATF4 signaling is increased at the final stage of HSV-1 replication, suggesting that HSV-1 disarms the unfolded protein response in the early stages of infection. HSV-1 may use ICP0 as a sensor to modulate the cellular stress response [146].

### 9.4. Stress Granules Formation

The formation of stress granules (SGs) is also involved in antiviral innate immune responses. In the case of virus infection, host cells may turn off the synthesis of intracellular protein translation by forming stress particles to resist virus replication [147]. SGs formation is related to inhibiting the host’s protein translation [148,149]. SGs contain a variety of components, such as untranslated mRNA, eukaryotic translation initiation factors (such as eIF4E, eIF4G, eIF4A, eIF2), and T-cell intracellular antigen 1 (TIA-1) and two markers of SGs: TIA-1-related protein (TIAR) and Ras GTPase activating protein-binding protein 1 (G3BP1) [147]. Phosphorylation of eIF2α (eIF2α-p) is also a landmark event in the formation of SGs [150]. eIF2α-p may lead to the retention of many translation initiation complexes in the cytoplasm and eventually induce the formation of SGs [151]. Xu et al. found that SGs induced by sodium arsenate (AS) and DL-Dithiothreitol (DTT) are blocked when the phosphorylation of eIF2α kinases double-stranded RNA-activated protein kinase (PKR) is significantly inhibited during PRV infection, suggesting that PRV-encoded protein itself or by recruiting some host factors to inhibit the formation of SGs, which will benefit PRV replication [152].

## 10. Conclusions and Prospects

PRV had caused great loss in the livestock industry. As an emerging and re-emerging infectious disease, it still poses a great threat to animal and human health. Like other herpes viruses, PRV can establish latent infection in specific tissues, which can lurk in the host’s nervous system for a long time. However, the detailed mechanisms are still not understood. Virus clearance in the body is closely related to the immune state of the host, especially innate immunity. Therefore, an in-depth understanding of the immune evade mechanism of PRV will benefit the development of effective PRV vaccines and antiviral drugs.

In this review, we summarized several strategies of PRV to escape the host’s innate immunity by disrupting important adaptor proteins in different signaling pathways, which are related to IFN production, IFN signaling pathway, inflammasome activation, apoptosis, autophagy, and ER stress. Although several PRV proteins have been reported to inhibit host innate immunity, the specific mechanisms for some proteins have not been clarified. For example, PRV EP0 could inhibit IFN response in primary cells, but it does not have this function in the nonhost cells [153]. PRV EP0 executes the different antiviral functions in different hosts is still unknown. In addition, HSV-1 ICP0, a homologous of PRV EP0, is an important protein involved in resisting host innate immunity [23,154]. Whether PRV EP0 has the same function is still unknown, which needs to be further investigated.

Although we have listed many natural immune escape strategies for PRV, compared with HSV-1 and VZV, the research on the regulation of host innate immunity by PRV needs to be further studied, such as NLR signaling pathway and inflammation. For example, AIM2 is a key DNA sensor for recognizing viral DNA upon DNA virus infection. PRV can infect swine and induce a strong inflammatory response. However, there is no AIM2 and its homolog in swine. Other host proteins maybe work as viral DNA sensors. We found that vimentin interacts with viral DNA and NLRP3, involved in the NLRP3 inflammatory response. Therefore, we proposed a new model that vimentin senses viral DNA and then recruits NLRP3 inflammasome to induce inflammatory responses in PRV-infected pigs.

## Figures and Tables

**Table 1:** NF-κB, cGAS-STING, and IFN signaling pathways are negatively regulated by PRV.

PRV Factors	Immune Elements and Mechanisms	Targeting Pathway	References
UL13	Degradation of PRDX1 Degradation of IRF3 Disrupts IRF3 binding to the IRF3-responsive promoter	cGAS-STING pathway cGAS-STING pathway cGAS-STING pathway	[49] [50] [51]
UL24	Degradation of IRF7 Degradation of p65 Inhibit the transcription of OASL Inhibit the transcription of ISG20	cGAS-STING pathway NF-κB signaling pathway IFN signaling pathway IFN signaling pathway	[52] [53] [54] [55]
US3	Degradation of IRF3 Degradation of Bclaf1	NF-κB signaling pathway IFN signaling pathway	[56] [57]
gE	Degradation of CBP/p300	NF-κB signaling pathway	[58]
UL50	Degradation of IFNAR1	IFN signaling pathway	[59]
UL42	Competes with ISG factor 3 (ISGF3) by binding to ISRE	IFN signaling pathway	[60]

## Data Availability

All data generated or analyzed during this study are included in this published article.
